# Flow-Synchronized Nasal Intermittent Positive Pressure Ventilation for Infants <32 Weeks' Gestation with Respiratory Distress Syndrome

**DOI:** 10.1155/2012/301818

**Published:** 2012-11-27

**Authors:** C. Gizzi, P. Papoff, I. Giordano, L. Massenzi, C. S. Barbàra, M. Campelli, V. Panetta, R. Agostino, C. Moretti

**Affiliations:** ^1^Neonatal Intensive Care Unit, Pediatric and Neonatal Department, “S.Giovanni Calibita” Fatebenefratelli Hospital, Isola Tiberina, Via Ponte Quattro Capi, 39-00186 Rome, Italy; ^2^Pediatric Emergency and Intensive Care, Department of Pediatrics, Policlinico “Umberto I,” Sapienza University of Rome, Viale Regina Elena, 324-00161, Rome, Italy; ^3^SeSMIT-A.Fa.R., Medical Statistics & Information Technology, Fatebenefratelli Association for Biomedical and Sanitary Research, Lungotevere de' Cenci, 5-00186 Rome, Italy; ^4^L'altrastatistica srl Consultancy & Training, Biostatistics Office, Via Ermino, 16-00174 Rome, Italy

## Abstract

*Aim*. To evaluate whether synchronized-NIPPV (SNIPPV) used after the INSURE procedure can reduce mechanical ventilation (MV) need in preterm infants with RDS more effectively than NCPAP and to compare the clinical course and the incidence of short-term outcomes of infants managed with SNIPPV or NCPAP. *Methods*. Chart data of inborn infants <32 weeks undergoing INSURE approach in the period January 2009–December 2010 were reviewed. After INSURE, newborns born January –December 2009 received NCPAP, whereas those born January–December 2010 received SNIPPV. INSURE failure was defined as FiO_2_ need >0.4, respiratory acidosis, or intractable apnoea that occurred within 72 hours of surfactant administration. *Results*. Eleven out of 31 (35.5%) infants in the NCPAP group and 2 out of 33 (6.1%) infants in the SNIPPV group failed the INSURE approach and underwent MV (*P* < 0.004). Fewer infants in the INSURE/SNIPPV group needed a second dose of surfactant, a high caffeine maintenance dose, and pharmacological treatment for PDA. Differences in O_2_ dependency at 28 days and 36 weeks of postmenstrual age were at the limit of significance in favor of SNIPPV treated infants. *Conclusions*. SNIPPV use after INSURE technique in our NICU reduced MV need and favorably affected short-term morbidities of our premature infants.

## 1. Introduction

Respiratory distress syndrome (RDS) is the single most important cause of morbidity and mortality in preterm infants. In past years, the standard treatment for this condition was endotracheal intubation and mechanical ventilation (MV), as well as exogenous surfactant therapy. Although life saving, MV is invasive, resulting in airway and lung injury and contributing to the development of bronchopulmonary dysplasia (BPD). Nasal continuous positive airway pressure (NCPAP) has been advocated to be a gentler form of respiratory support that makes it possible to reduce the need for MV in preterm infants [1–3]. NCPAP combined with early surfactant replacement therapy, administered by intubation and rapid extubation (intubation-surfactant-extubation, INSURE), has been introduced as a primary mode of respiratory support in premature infants with RDS with a varying degree of success, depending on patients' gestational age (GA) and the severity of the radiological stage of RDS and FiO_2_ at surfactant administration [4–10]. An evidence-based review showed that surfactant given at an early stage of RDS with extubation to NCPAP, compared with surfactant given later and continued MV, is associated with a reduced need for MV, a lower incidence of BPD and fewer air-leak syndromes [11].

Nasal intermittent positive pressure ventilation (NIPPV) is a noninvasive mode of ventilation that offers more ventilatory support than NCPAP. NIPPV may be synchronized (SNIPPV) or nonsynchronized to the infant's breathing efforts. Several observations favor SNIPPV. Kiciman et al. [12] demonstrated that thoracoabdominal motion asynchrony and flow resistance through the nasal prongs decreased in neonates on SNIPPV, with improved stability of the chest wall and pulmonary mechanics. Moreover, delivering the peak inspiratory pressure immediately after the start of a respiratory effort, when the glottis is open, allows pressure to be effectively transmitted to the lungs, with little or no deviation through the esophagus into the stomach, obtaining the double advantage of increasing tidal volume (Vt) and reducing the risk of gastrointestinal side effects. In doing so, it is also possible that SNIPPV recruits collapsed alveoli and increases functional residual capacity (FRC). Recently, Owen et al. [13] described that during nonsynchronized NIPPV, Vt increases only when pressure peaks occur during spontaneous inspiration, suggesting that synchronization may be beneficial. In a previous study our group reported that application of SNIPPV was associated with increased tidal and minute volumes and decreased respiratory effort when compared with NCPAP in the same infant [14]. Finally, Aghai et al. [15] and Chang et al. [16] demonstrated that infants receiving SNIPPV have decreased work of breathing (WOB). Asynchronous breaths, on the contrary, may increase the risk of pneumothorax (PNX), blood pressure and cerebral blood flow velocity fluctuations and WOB [16, 17]. 

The purpose of our study was to evaluate whether in premature infants (GA < 32 wks) with RDS, SNIPPV used as ventilatory support immediately after surfactant administration using the INSURE technique is effective in further reducing the incidence of MV within the following 72 hours when compared to the conventional INSURE/NCPAP treatment. Our aim was also to compare the NICU clinical course and the incidence of short-term outcomes of pretem infants managed with SNIPPV or NCPAP after INSURE.

## 2. Patients and Methods

Chart data from inborn preterm infants with GA < 32 weeks admitted to our NICU from January 2009 to December 2010 were reviewed retrospectively with the aim of identifying newborns treated with nasal ventilation and the INSURE approach. Management of RDS before INSURE and the INSURE procedure were similar for all infants. Spontaneously breathing preterm newborns, not requiring intubation at birth for cardiopulmonary resuscitation, received early rescue NCPAP if chest retractions and/or grunting and/or tachypnea and/or oxygen need were present. Infants were routinely treated with caffeine (loading dose of 20 mg per kg of caffeine citrate followed by a daily maintenance dose of 5 mg per kg; daily maintenance dose could be doubled to 10 mg per kg in case of persistent apnoeic spells). Intubation for surfactant administration (INSURE technique) was performed if the FiO_2_ requirement on NCPAP (CPAP level 5-6 cm H_2_O) was >0.4 for more than 30 min, to maintain transcutaneous oxygen saturation between 85 and 93% in the presence of radiological signs of RDS. Poractant alfa (Curosurf-Chiesi Farmaceutici, Parma, Italy) 200 mg/kg was given endotracheally, followed by manual ventilation by bag for 2–5 min. A preterm size self-inflating ventilation bag was used for the procedure. During manual ventilation, titration of O_2_ delivery was achieved by connecting the inlet of the self-inflating bag to an air/oxygen blender. Pressure was controlled by an attached disposable manometer. Pain control for elective endotracheal intubation was obtained by administering fentanyl 0.5–2 mcg/kg 5–10 min before intubation. After surfactant and manual ventilation, in the presence of a good respiratory drive and a satisfactory transcutaneous oxygen saturation value, infants were extubated. To reverse the potential respiratory depression because of opioids, infants without a good respiratory drive could receive a single dose of Naloxone (0.04 mg/kg). After extubation, infants referred from January to December 2009 (INSURE/NCPAP historical control group) were treated with ventilator-derived NCPAP (V.I.P. Bird Gold ventilator-Viasys Healthcare, Yorba Linda, CA, USA) as per standard protocol, while infants referred from January to December 2010 (INSURE/SNIPPV study group) were treated with flow-SNIPPV (“Giulia” Neonatal Nasal Ventilator-Ginevri Medical Technologies, Rome, Italy) according to a new institutional protocol for RDS. The device synchronizes NIPPV by means of a pneumothacograph interposed between the nasal prongs and the Y piece [18]. Before that time, in our unit, SNIPPV was mostly used to help infants weaning from MV after extubation and to treat apnoea of prematurity.

Nasal prongs of the same type (Ginevri Medical Technologies, Rome, Italy) were used for both ventilation modes. The size of the prongs was determined by the infant's weight. The largest possible prongs were used, with a snug fit to avoid leakage. No precautions were taken to avoid leakage from the mouth. 

Mechanical ventilation was started in case of INSURE failure defined as: (1) FiO_2_ > 0.4 to maintain SpO_2_ 85–93%; (2) significant apnoea defined as more than 4 episodes of apnoea/hour or more than 2 episodes of apnoea/hour if bag and mask ventilation were required; (3) respiratory acidosis defined as pCO_2_ > 65 mmHg (8.5 kPa) and pH < 7.20 on arterial or capillary blood gas.

A second dose and additional doses of surfactant of 100 mg/kg could be administered to infants who were on MV, while a second INSURE was never tried. All infants were started on parenteral nutrition (PN) within the first 24 h of life, with dextrose, amino acids, and lipids. Total fluid volumes were increased daily until a goal of 140 to150 mL/kg/day was achieved during the first week of life. Infants were started on trophic feeds when clinically stable. Enteral nutrition was increased by 10–20 mL/kg every day as tolerated until a goal of 150 mL/kg/day. PN was stopped when full feeds (120 mL/kg/day) were tolerated.

Echocardiography was performed in all infants at 24–72 h of life and intravenous treatment with ibuprofen or indomethacin for a patent ductus arteriosus (PDA) was based on echocardiography and clinical signs. Cerebral echography was performed within the first 48 h of life, repeated at 7 days, and then every week until discharge. Intracranial hemorrhages (IVH) were classified as described by Volpe [19] and periventricular leucomalacia (PVL) as described by de Vries et al. [20]. Late-onset sepsis was diagnosed when a positive blood culture occurred in a sick infant after the first 72 hours of life. Necrotizing enterocolitis (NEC) was classified based on Bell's criteria [21]. Retinopathy of prematurity (ROP) grades I-V were defined as per international classification [22]. The INSURE/SNIPPV group was compared with the INSURE/NCPAP group to evaluate whether SNIPPV reduced the need for MV in the 72 hours after INSURE. The two groups were also compared in terms of incidence of air leaks, need for a second dose of surfactant, need for a high maintenance dose of caffeine, need for postnatal steroids, O_2_ dependency at 28 days and 36 weeks of postmenstrual age (PMA), late-onset sepsis, nasal complications, feeding intolerance, NEC, PDA, IVH, PVL, ROP, and death. Duration of MV for infants who failed the INSURE approach, days on nasal ventilation, days on oxygen, days on parenteral nutrition, and length of hospital stay were also evaluated in the two study groups.

The maternal variables examined included type of delivery, antenatal steroid treatment, pregnancy induced hypertension, prolonged premature rupture of membranes (pPROM) > 18 h, placental abruption, intrauterine growth restriction (IUGR), and clinical chorionamnionitis (defined as the presence of fever with one or more of the following: maternal leukocytosis > 15,000/mm^3^, uterine tenderness, fetal tachycardia, foul-smelling amniotic fluid).

Approval for this study was obtained from the Ethics Committee of the “S. Giovanni Calibita” Fatebenefratelli Hospital, Isola Tiberina, Rome.

## 3. Statistical Methods

All data were collected in an Excel database and analyzed using the statistical package STATA 12.0. Continuous normally distributed variables were compared using the *t* Student test for unpaired data and categorical variables were compared using the chi-square test. The Mann-Whitney test was performed to compare continuous variables that were not normally distributed. The Shapiro Wilk test was used to evaluate normally distributed assumptions.

A *P* value less than 0.05 was considered statistically significant.

## 4. Results

One hundred and sixty-seven infants with GA < 32 weeks were referred to our NICU in the 2 study periods. Surfactant was administered to 101 infants (60.5%); 64 of them underwent INSURE treatment (31 newborns in the INSURE/NCPAP historical control group and 33 in the INSURE/SNIPPV study group) and were included in this review. Characteristics of the newborns included in the 2 groups did not demonstrate significant differences (Table 1). In particular, Clinical Risk Index for Babies (CRIB) scores [23, 24], radiographic classification [25], FiO_2_ values, and transcutaneous PCO_2_ (tcPCO_2_) values before surfactant treatment indicated that the RDS severity was similar for the 2 groups. After INSURE, infants in the historical group received NCPAP at a pressure level of 5-6 cm H_2_O with a flow rate of 8.0 ± 0.5 L/min while infants in the study group received SNIPPV in the assist/control mode (i.e., ventilator assisting each spontaneous breath) with the following initial respiratory parameters: Ti 0.32 ± 0.02 sec, back-up rate 35 ± 5 bpm, PIP 15 ± 2 cm H_2_O, PEEP 5.5 ± 0.5 cm H_2_O, flow rate 8.0 ± 0.5 L/min. 

Table 2 reports neonatal outcomes in the 2 study groups. Eleven (GA 27.9 ± 1.7 weeks, BW 1056 ± 222 g) out of 31 infants in the INSURE/NCPAP group versus 2 (GA 25 and 27 weeks, BW 670 and 1080 g, resp.) out of 33 infants in the INSURE/SNIPPV group met the INSURE failure criteria and underwent endotracheal MV (35.5% versus 6.1%; *P* = 0.004). Failure was due to pneumothorax in 1 infant, intractable apnoea in 4 infants, hypercapnia in 3 infants, and increased oxygen requirement in 3 infants in the INSURE/NCPAP group, while in the INSURE/SNIPPV group both infants failed because of increased oxygen requirement. 

INSURE failure occurred at median (range) 48.1 (5–71) hours after surfactant administration in the INSURE/NCPAP group and at 9.5 (6–13) hours in the INSURE/SNIPPV group. Six hours after surfactant administration, the FiO_2_ requirement for infants still on nasal ventilation was significantly higher in the INSURE/NCPAP group (median (range): 0.30 (0.21–0.45) versus 0.22 (0.21–0.40); *P* < 0.001). More infants in the INSURE/NCPAP group needed a second dose of surfactant (22.6% versus 3%; *P* = 0.025) and a high maintenance dose of caffeine (29% versus 9.9%; *P* = 0.041). Treated PDA was also more frequent in the INSURE/NCPAP group (25% versus 6.5%; *P* = 0.041). Among the 10 patients with treated PDA, only 1 in the INSURE/NCPAP group required surgical ligation. Four (2 per group) of those infants who were successfully treated with the INSURE approach subsequently needed MV due to late-onset sepsis at median age of 13 days (range 8–21 days). Although fewer infants in the INSURE/SNIPPV group were O_2_ dependent at 28 days and 36 weeks PMA, this was at the limit of statistical significance. Other neonatal outcomes did not differ in the 2 groups, as reported in Table 2. Nasal complications, including columella nasi bleeding, flaring of the nostrils, and snubbing of the nose, were all transient, and the incidence was similar in the two groups. Some infants in both groups had moderate abdominal distention; however the incidence of feeding intolerance was similar in the two groups. One infant in the INSURE/NCPAP group developed NEC (Bell stage IIA) by day 20 of life. The observed ROP case was grade I. NICU course did not differ between groups (Table 3).

## 5. Discussion 

Early surfactant therapy administered by INSURE technique and combined with NCPAP has been applied in preterm infants with RDS to prevent ventilator-associated lung injury. This strategy is effective in improving respiratory outcome and reducing the need for MV, although several studies reported an INSURE/NCPAP approach failure ranging between 26% and 50% [10]. In our pre-SNIPPV period, INSURE failure occurred in about 35% of infants <32 weeks' gestation, similar to literature reports. The introduction of flow-SNIPPV in our Unit and its use after INSURE significantly reduced the need for MV. 

We previously observed that flow-SNIPPV in the post extubation period supports respiratory effort more effectively than NCPAP [14, 18]. According to present data, flow-SNIPPV also seems to be promising in treating infants in the acute phase of RDS, as a primary mode of ventilation after INSURE. 

Compared with NCPAP, SNIPPV improves ventilation by increasing Vt [13, 14] and decreasing respiratory effort [12, 14–16], thus representing an ideal mode of noninvasive support. These mechanisms of action probably account for the higher success of the INSURE/SNIPPV strategy over the classical INSURE/NCPAP reported in our study. Indeed, in our series of preterm infants the prominent effects of flow-SNIPPV were those of augmenting and stimulating spontaneous breathing as demonstrated by the absence of respiratory acidosis and apnoeic episodes as reasons for failure in infants who received this mode of ventilation. 

It has been observed that SNIPPV significantly reduces PCO_2_ values when compared with NCPAP in preterm infants [14, 26] as a consequence of improved alveolar ventilation. Moreover, apnoeic spells are common in premature infants and are recognized as a significant reason for MV use. Barrington et al. [27] found a trend towards a reduction of apnoeic episodes per day in infants treated with SNIPPV after extubation, while Lin et al. [28] suggested that synchronization may increase the success of NIPPV in reducing apnoeic spells. Conversely, Ryan et al. [29] and Pantalitschka et al. [30] found that NIPPV offers no advantages over NCPAP in treating apnoea of prematurity. According to these observations, fewer infants in the INSURE/SNIPPV group needed a high maintenance dose of caffeine for persistent apnoeic spells and no infant underwent MV due to apnoea in this group. Synchronized NIPPV may effectively help preterm infants suffering apnoeic episodes to counteract the mechanisms that contribute to this pathology better than NCPAP and NIPPV. 

FiO_2_ requirement 6 hours after INSURE was lower in SNIPPV treated infants. One possible explanation for this association is that SNIPPV, favoring alveolar recruitment and keeping the lung open by applying a higher mean airway pressure, may prevent RDS worsening more effectively than NCPAP. For the same reasons, SNIPPV probably helps exogenous surfactant distribution in the lungs and its more effective action, as suggested by a reduced need for a second surfactant dose in this group.

Recently, 3 randomized controlled trials studied the effects of NIPPV applied in the acute phase of RDS as a primary mode of respiratory support before surfactant replacement. Kugelman et al. [31] observed that NIPPV, compared with NCPAP, decreased the requirement for endotracheal ventilation in premature infants <35 weeks with RDS, and this was associated with a reduced incidence of BPD. In this study however the INSURE approach was not used. Sai Sunil Kishore et al. [32] used NIPPV at the first signs of RDS, and coupled this technique with the INSURE approach in premature infants with GA ≥ 28 weeks. Similar to our reports, they found that the need for intubation and MV was lower with NIPPV. Finally, Meneses et al. [33] could obtain similar results using NIPPV only in infants weighing > 1000 g. According to these reports, nonsynchronized early NIPPV seems to be beneficial for slightly older and heavier infants when compared with NCPAP. However, as SNIPPV presents potential advantages over NCPAP and NIPPV, its use soon after birth in preterm infants <1000 g deserves further investigation. 

A significant reduction in BPD has been reported when NIPPV is used as respiratory support after extubation or as a primary mode for RDS [34–37]. In our study, the difference in O_2_ dependency at 28 days and 36 weeks between the two groups was at the limit of statistical significance, probably due to the small number of patients included. As MV in the first days of a preterm infant is a major factor for BPD [38, 39], avoiding endotracheal tube ventilation remains of paramount importance in preventing ventilator-induced lung injury. 

RDS and PDA are common comorbidities in premature infants. In our series, more infants in the INSURE/NCPAP group needed pharmacological treatment for PDA. Symptomatic PDA is an identified risk factor for INSURE failure [40] and may have contributed to the higher need for MV in this group. Moreover, as mechanical ventilation strategies may influence ductal closure [41], whether flow-SNIPPV may have a direct effect on PDA should be investigated further.

In our study a second INSURE after the first INSURE failure was not attempted. Recently, Dani et al. [42] found that multiple INSURE procedures were followed by a similar respiratory outcome to the single procedure in a cohort of extremely premature infants. Whether the multiple INSURE approach might be a useful alternative to surfactant given during MV requires specific studies.

Abdominal distension is commonly observed in infants undergoing nasal ventilation. Although mild abdominal distension usually causes no severe complications, it may play a role either in delaying the speed of oral feeding or in reducing the efficacy of ventilation. In our series, one infant per group had to discontinue oral feeding for 24 hours while on nasal ventilation, while 2 infants in the INSURE/NCPAP group and 3 infants in the INSURE/SNIPPV group delayed the daily increase of oral feeds. Overall, days on parenteral nutrition were similar in the two groups. Nasal complications were also observed, but not serious enough to cause ventilation to be suspended. 

The incidence of cerebral damage was very low in the whole group, confirming the safety of this ventilatory approach in a population of relatively large preterm infants. Moreover, the two study groups did not differ in terms of short-term outcomes at discharge, suggesting that flow-SNIPPV could provide effective as well as safe ventilatory support for the treatment of infants with RDS after surfactant treatment.

The main limitations of this study are in the retrospective design and in the small number of patients included. Although this is a small retrospective study, it has been conducted over a relatively short clinical period, during which we are not aware of any significant shift in clinical practice other than the introduction of flow-SNIPPV as a respiratory support after INSURE, therefore we do not believe that other changes in clinical practice not recorded for this analysis might explain the differences between the two groups. Nevertheless, these data need to be confirmed in a randomized controlled trial, and the possible underlying protective mechanisms of SNIPPV on acidosis and/or on apnoea deserve specific investigation. 

Another limitation of our study relates to the small number of infants included weighing less than 1000 g at birth, since most of the difficulties in keeping infants away from MV are encountered with these tiny newborns. In our series, 5 infants in each group had a birth weight <1000 g. Among these, 4 in the NCPAP group and 1 in the SNIPPV group failed the INSURE approach. Thus, further work is needed to establish the effectiveness of SNIPPV in extremely low birth weight infants.

## 6. Conclusions

Our data suggest that, for infants being treated with nasal ventilation for RDS, the use of flow-SNIPPV after surfactant administration using the INSURE technique is safe and beneficial, as evidenced by the decreased need for MV, with no worsening of prematurity-related outcomes, compared with infants who underwent the classical INSURE/NCPAP approach. Further studies should be conducted to confirm these findings, to evaluate the real improvement in long-term outcome in SNIPPV treated newborns, to determine the optimal ventilatory settings of the SNIPPV system and to investigate the possibility of using SNIPPV to treat apnoea of prematurity and as a primary support for idiopathic RDS before surfactant administration particularly in extremely low birth weight infants.

## Figures and Tables

**Table 1:** Neonatal characteristic in the two study groups.

	INSURE/NCPAP (n.31)	INSURE/SNIPPV (n.33)	*P* value
Gestational age (wks)	29.1 ± 1.4	28.7 ± 1.3	0.768
Birth weight (g)	1305 ± 364	1283 ± 278	0.786
M/F	14/17	13/20	0.641
Multiple births	7 (22.6)	8 (24.2)	0.085
Antenatal steroids	27 (87.1)	26 (78.8)	0.689
Main maternal pregnancy diseases			
(i) hypertensive disorders	6 (19.3)	9 (27.2)	0.455
(ii ) pPROM	6 (19.3)	5 (15.1)	0.729
(iii) placental abruption	4 (12.9)	3 (9.1)	0.704
(iv) corionamnionitis	3 (9.6)	4 (12.1)	1.000
(v) IUGR	5 (16.1)	5 (15.1)	1.000
Cesarean section	27 (87.0)	28 (84.8)	0.796
Apgar score at 5′	8 (5–9)	8 (6–9)	0.947
CRIB score	2 (0–11)	1 (0–8)	0.078
RDS moderate to severe*	20 (64.5)	23 (69.7)	0.625
Age at NCPAP (min)	30 (15–120)	30 (15–120)	0.994
Age at INSURE (hours)	4 (0.5–17)	4 (0.5–23)	0.736
FiO_2_ at INSURE	0.44 ± 0.05	0.43 ± 0.03	0.332
tcPCO_2_ at INSURE (mm Hg)	46.6 ± 6.6	48.6 ± 7.9	0.278

Values are given as mean ± SD, median (range), or number and (%).

*Radiographic classification.

**Table 2:** Neonatal outcomes in the two study groups.

	INSURE/NCPAP (n.31)	INSURE/SNIPPV (n.33)	*P* value
INSURE failure	11 (35.5)	2 (6.1)	0.004
Pneumothorax	1 (3.2)	0	0.484
Surfactant second dose	7 (22.6)	1 (3.0)	0.025
Caffeine high maintenance dose	9 (29.0)	3 (9.9)	0.041
PDA treated	8 (25.8)	2 (6.1)	0.041
Postnatal steroids	4 (12.9)	1 (3.0)	0.190
O_2_ dep. at 28 days	6 (19.3)	1 (3.0)	0.050
O_2_ dep. at 36 weeks PMA	4 (12.9)	0	0.050
Late onset sepsis	4 (12.9)	4 (12.1)	1.000
Feeding intolerance	3 (9.7)	4 (12.1)	1.000
NEC	1 (3.2)	0	0.484
IVH (1-2°)	2 (6.4)	2 (6.0)	1.000
IVH (3-4°)	1 (3.2)	1 (3.0)	1.000
PVL	0	0	1.000
ROP	0	1 (3.0)	1.000
Death	0	0	1.000

Values are given as number and (%).

**Table 3:** NICU course in the two study groups.

	INSURE/NCPAP (n.31)	INSURE/SNIPPV (n.33)	*P* value
Duration of MV* (h)	29 ± 21	39 ± 22.8	0.073
Days on nasal ventilation	4.8 (1–62)	4.9 (1–25)	1.000
Days on oxygen	7.4 (1–62)	6 (1–35)	0.704
Days on parenteral nutrition	13.2 ± 8.2	15.6 ± 9.8	0.294
Length of hospital stay (days)	49 ± 19	48 ± 25	1.000

Values are given as mean ± SD or median (range).

*For infants who failed INSURE approach.
